# Effects of Irrigation Regime and Seasonal Dynamics on the Rhizosphere Microbiome on Calcaric Cambisols in Western Iberian Vineyards: An Observational Study Across Seasons

**DOI:** 10.3390/plants15213468

**Published:** 2026-11-06

**Authors:** Luis Francisco Fernández-Pozo, María Angeles Rodríguez-González

**Affiliations:** Environmental Resources Analysis Research Group, University of Extremadura, 06006 Badajoz, Spain; marodgon@unex.es

**Keywords:** rhizosphere microbiome, 16S/ITS metabarcoding, irrigation management, alpha and beta diversity, soil sustainability, biotic homogenization

## Abstract

Understanding how management and seasonality are associated with the soil microbiome is relevant for the sustainability of Mediterranean viticulture. This observational study compared the rhizosphere microbiome of rainfed and irrigated ‘Pardina’ vineyards on Calcaric Cambisols (Tierra de Barros, Extremadura, Spain) in five sampling campaigns (spring 2024–spring 2026) using 16S rRNA gene and ITS1 amplicon metabarcoding. The 30 soil samples were aggregated into ten community profiles per marker (five campaigns × two management), so inference is exploratory and limited by the absence of plot-level replication. Fungal communities in irrigated vineyards showed lower observed genus richness (median 180 vs. 297), Shannon diversity (3.05 vs. 3.84) and evenness than rainfed ones, with the same direction in all five campaigns, and a tendency toward higher dominance of a few genera (Berger–Parker 19.3% vs. 11.9%; not significant after correction), whereas prokaryotic richness and Shannon diversity did not differ consistently. Bray–Curtis PERMANOVA indicated a modest association between management and prokaryotic composition (*R*^2^ = 0.237, *p* = 0.048), which was not robust to permutations restricted within campaigns, and no detectable association for fungi (*R*^2^ = 0.151, *p* = 0.269); both results were sensitive to the inclusion of the spring 2026 campaign. The season and its interaction with management were not significant. Soil pH, available phosphorus and organic carbon differed between managements and planting density differed by design, so the patterns cannot be attributed to irrigation itself. The bacteria-to-fungi ratio, a sequencing-derived index, is reported descriptively only. These results identify candidate taxa and hypotheses to be tested in replicated experiments that include soil moisture and functional measurements.

## 1. Introduction

The rhizosphere microbiome represents one of the most complex and dynamic biological interfaces in the agrosystem, playing a critical role in nutrient cycling, plant nutrition and growth, the suppression of plant pathogens, and the maintenance of soil structure [1,2]. Roots actively select a subset of the soil microbial reservoir (species sorting), shaping microbial communities that have a direct impact on abiotic stress tolerance and host defense [3,4]. In Mediterranean and semi-arid agrosystems, this interaction takes on particular importance, given that water availability is the primary limiting factor for primary productivity.

In grapevines (*Vitis vinifera* L.), the edaphic and rhizosphere microbiome not only influences the crop’s phytosanitary status and productivity but also modulates metabolic processes that affect the organoleptic composition of grapes and wine [3,5]. This link has solidified the concept of microbial terroir, which posits that microbial communities are part of the vineyard’s biogeographic identity at the continental, regional, and local scales [6,7,8,9]. However, the architecture of the microbial terroir does not respond exclusively to spatial gradients but rather emerges from the hierarchical interaction between soil physicochemical properties, climatic conditions, phenology, plant genotype, and agronomic management [9,10,11].

Soil properties are established as primary determinants of alpha and beta diversity in the microbiome [5,11,12]. In vineyard soils, variables such as pH, soil organic carbon (SOC), the C:N ratio, available phosphorus, and texture explain a significant fraction of community variance [5,13]. While in fungal communities, environmental filtering by pH and organic matter may prevail even at fine spatial scales [14] in bacterial communities, carbon availability is typically associated with an increase in species richness and niche breadth [11]. This framework is critical in Calcaric Cambisols soils, where the chemistry of calcium and carbonates (CaCO_3_) restricts the dynamics of organic carbon and alters the bioavailability of nutrients [15]. In these alkaline environments, the presence of calcareous matrices favors the shift of oligotrophic taxa toward copiotrophic clades and affects total microbial biomass [16,17,18].

In addition to edaphic selectivity, there is anthropogenic disturbance resulting from changes in the management. The conversion of traditional rain-fed vineyards to irrigated ones has improved production stability, but its ecological implications for the rhizosphere microbiota remain a subject of debate [6,13]. Although it has been hypothetically assumed that irrigation increases microbial richness by alleviating water limitations, empirical evidence suggests that moisture saturation reduces niche heterogeneity, promotes community convergence, and selects for fast-growing opportunistic taxa [9,11]. Consequently, the response of the rhizosphere microbiota to irrigation may involve changes in community assembly and beta diversity rather than a simple increase in local alpha diversity [19].

Additionally, seasonality introduces a dynamic intra-annual component. Fluctuations in temperature, soil moisture, and root exudation patterns throughout the growing season impose temporal variations on the rhizosphere microbiome [10,20]. These seasonal dynamics affect the different microbial kingdoms asymmetrically: fungal communities generally exhibit greater sensitivity to climatic factors, seasonality, and macroscale terroir, whereas bacterial communities show a closer coupling with pH, available carbon, and agronomic management variables [8,21]. Dominant prokaryotic taxa such as Proteobacteria, Actinobacteria, and Acidobacteria, as well as fungal clades belonging to Ascomycota, Basidiomycota, and genera such as Mortierella, Alternaria, or Solicoccozyma, respond differently to these environmental gradients [5,6]. This differential response justifies the dual metabarcoding approach (16S rRNA gene and ITS region) to comprehensively assess community restructuring and the dynamics of key functional groups (e.g., nitrogen fixers such as Rhizobiaceae, nitrifiers such as Candidatus Nitrososphaera, or phytopathogenic taxa such as Fusarium) [7,22].

Despite advances in the characterization of the viticultural microbiome, knowledge gaps remain regarding the association between the management (rainfed vs. irrigated) and seasonality in Mediterranean agroecosystems established on Calcaric Cambisols. The objective of this observational study was to compare the alpha diversity, beta diversity, and taxonomic composition of the prokaryotic (Bacteria and Archaea; 16S rRNA gene) and fungal (ITS1) communities in the rhizosphere of *Vitis vinifera* L. in rainfed and irrigated vineyards sampled in spring and autumn campaigns during 2024–2026. Two exploratory working hypotheses were considered: (i) differences in the rhizosphere microbiota associated with management would be more pronounced in community composition (beta diversity) than in local richness (alpha diversity); and (ii) prokaryotic and fungal communities would respond differently to management and season, with the fungal microbiota showing greater sensitivity to seasonal dynamics. Because the design comprises a limited number of vineyards and campaigns, and the management is partly confounded with planting density and soil properties, the results are interpreted as associations under the conditions studied and not as causal effects of irrigation.

## 2. Results

### 2.1. Soil Physicochemical Properties

The physicochemical properties of the vineyard soils are summarized in Table S1 (Supplementary Materials) as means ± standard deviations by management and by season. Soils were alkaline (pH 8.14 ± 0.77 in rainfed and 7.65 ± 0.86 in irrigated vineyards), non-saline (electrical conductivity 0.20 ± 0.08 and 0.32 ± 0.27 dS m^−1^), sand-dominated (sand 63.8–65.0%, silt 19.2–19.8%, clay 15.8–16.4% across groups) and poor in organic carbon (0.74 ± 0.26% and 0.99 ± 0.33%) and total nitrogen (0.055 ± 0.020% and 0.070 ± 0.020%). The largest contrasts between rainfed and irrigated soils were found for available phosphorus (21.3 ± 5.8 vs. 48.1 ± 25.6 ppm), carbonates (3.58 ± 5.03% vs. 1.82 ± 1.07%), exchangeable Mg (1.71 ± 0.79 vs. 2.46 ± 0.95 meq 100 g^−1^) and K (0.64 ± 0.28 vs. 0.86 ± 0.32 meq 100 g^−1^), and bulk density (1.68 ± 0.23 vs. 1.54 ± 0.17 Mg m^−3^), although the standard deviations were large and overlapped. Differences between spring and autumn means were small for all properties. The soil data are aggregated by management and by season; plot- and date-specific values and soil moisture are not available. The association between soil properties and community composition could therefore not be tested formally, and the contrasts above are treated as potential confounders of the rainfed/irrigated comparison (see Section 3).

### 2.2. Alpha Diversity of the Rhizosphere Microbiome

**Figure 1:**
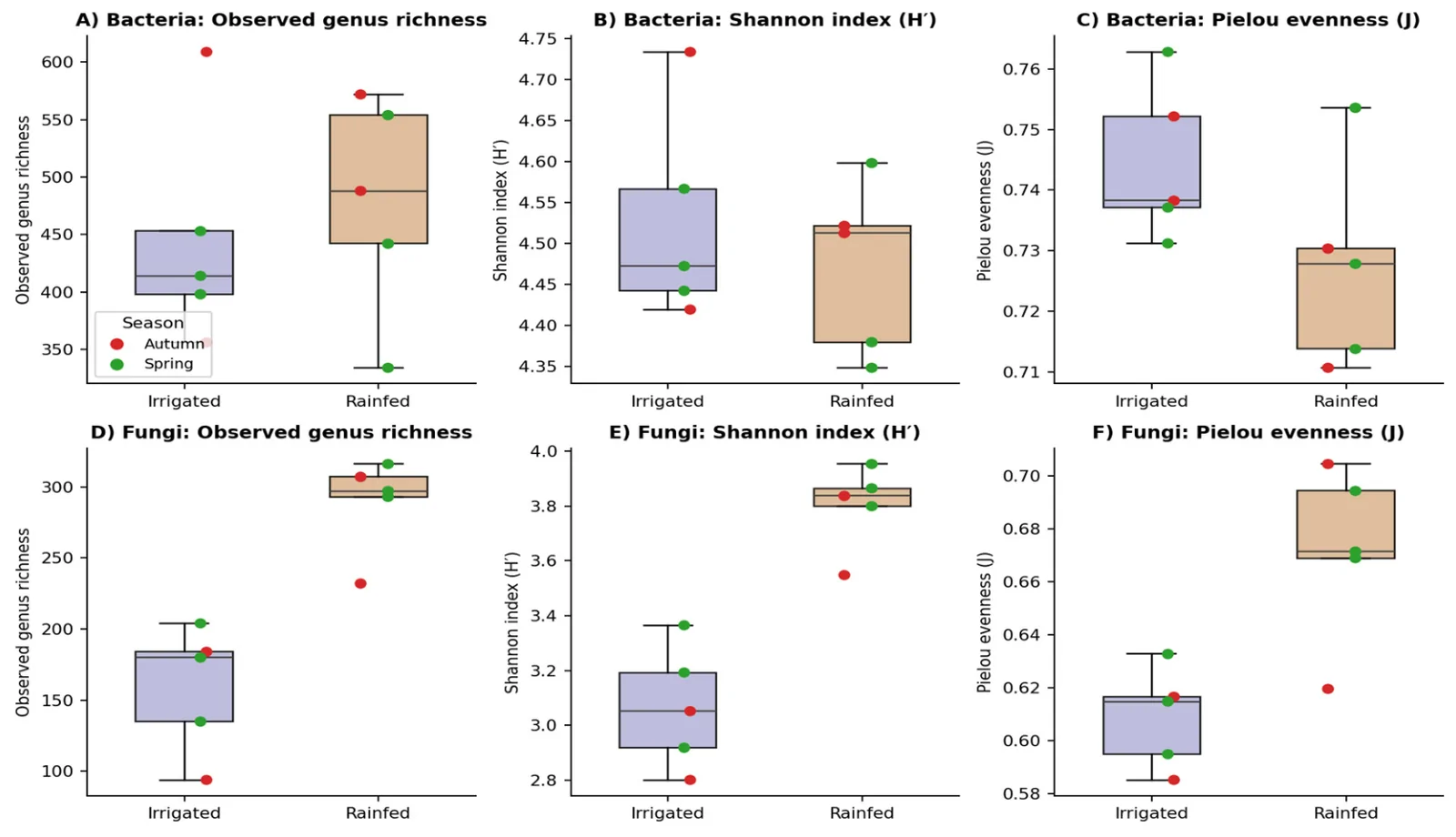
**Alpha diversity of the rhizosphere prokaryotic and fungal communities in irrigated and rainfed vineyards.** Box plots (median, interquartile range; whiskers extend to 1.5 × IQR) of (**A**,**D**) observed genus richness, (**B**,**E**) Shannon index (*H*′, natural logarithm) and (**C**,**F**) Pielou’s evenness (*J*) for prokaryotes (**A**–**C**; 16S rRNA gene) and fungi (**D**–**F**; ITS1) under irrigated (purple) and rainfed (tan) management. Each point is one campaign × management community profile (*n* = 5 per box), colored by season (red, autumn; green, spring).

Alpha diversity was evaluated at the genus level for each of the ten campaign × management community profiles per marker (Table 1; Figure 1). In the prokaryotic community (Bacteria and Archaea), observed genus richness (median 488 [interquartile range, IQR 442–554] in rainfed vs. 414 in irrigated vineyards) and Shannon diversity (4.51 vs. 4.47) did not differ consistently between managements: the 95% confidence intervals of the paired differences (irrigated/rainfed) included zero (−32 genera, 95% CI −137 to 73; Δ*H*′ = 0.05, −0.15 to 0.26), and irrigated richness was lower in only three of the five campaigns. Pielou’s evenness was slightly higher in irrigated than in rainfed vineyards in all five campaigns (median 0.738 vs. 0.728; Δ*J* = 0.017, 95% CI 0.004 to 0.030), consistent with a lower Gini coefficient (0.812 vs. 0.848), although these differences were no longer significant after Holm correction (*p* = 0.102 and *p* = 0.118).

Fungal communities showed larger and more consistent differences. Irrigated vineyards had lower observed genus richness (median 180 vs. 297), Shannon diversity (3.05 vs. 3.84) and Pielou’s evenness (0.615 vs. 0.671) than rainfed ones, in all five campaigns (Figure 1D–F). The mean paired differences were −130 genera (95% CI −163 to −96), Δ*H*′ = −0.73 (−1.09 to −0.38) and Δ*J* = −0.063 (−0.100 to −0.026), and remained significant after Holm correction (*p* = 0.002, *p* = 0.019 and *p* = 0.027, respectively). Berger–Parker dominance tended to be higher in irrigated soils (median 19.3% vs. 11.9%; *p* = 0.095 after correction), whereas the Gini coefficient did not differ clearly (0.880 vs. 0.858). Excluding the spring 2026 fungal profiles, whose taxonomic assignment was incomplete (Methods 4.5), gave the same direction in all four remaining campaigns (richness lower in irrigated soils by 140 genera on average; Table S4).

### 2.3. Beta Diversity and Structure of Microbial Communities

**Figure 2:**
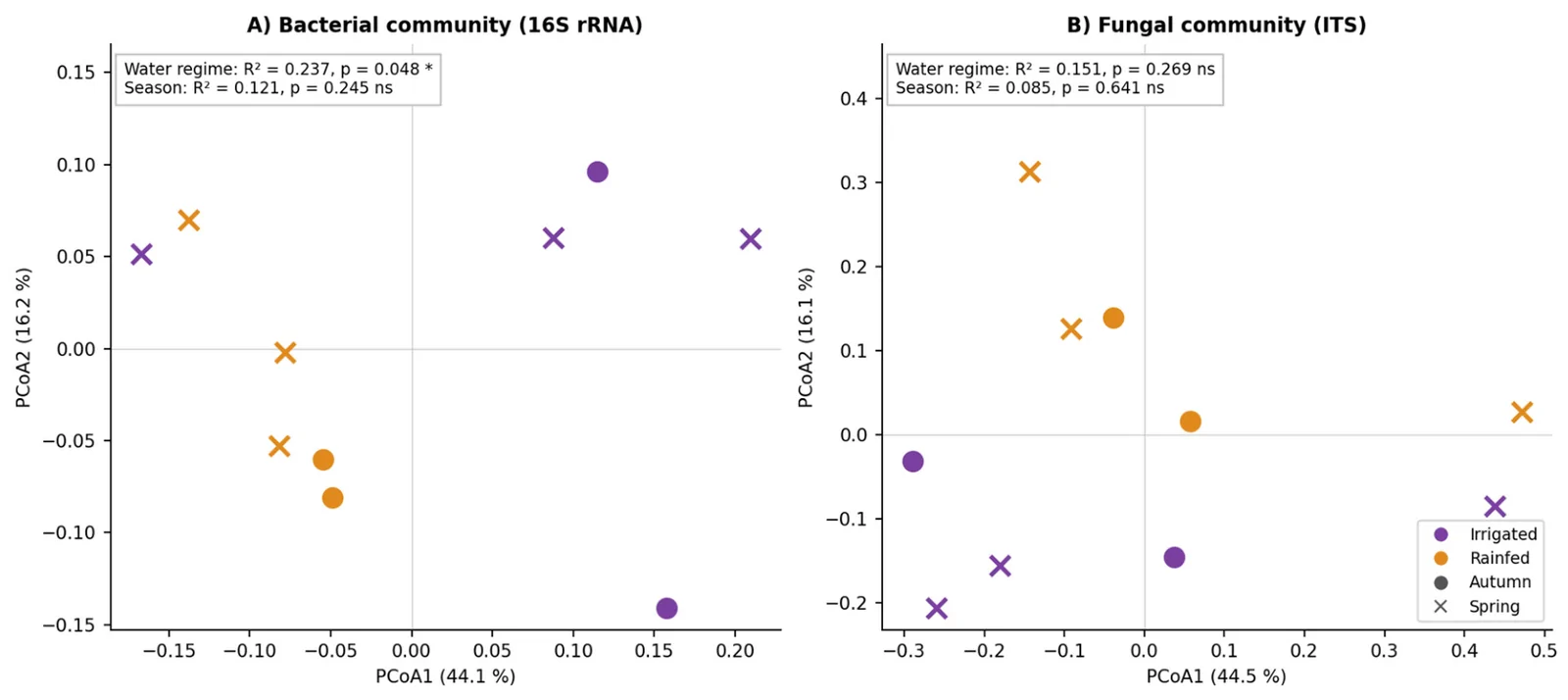
**Principal coordinates analysis (PCoA) of Bray–Curtis dissimilarities at genus level.** (**A**) Prokaryotic community (16S rRNA gene) and (**B**) fungal community (ITS1). Point colors indicate management (purple, irrigated; orange, rainfed) and point shapes indicate season (circles, autumn; crosses, spring). Each point is one campaign × management profile (*n* = 10 per panel). PERMANOVA results (Table 2) are annotated in each panel (*, *p* < 0.05; ns, not significant); for panel (**A**), the management term was not significant (*p* = 0.125) when permutations were restricted within campaigns.

Bray–Curtis dissimilarities between the rainfed and irrigated profiles of the same campaign ranged from 0.20 to 0.34 for prokaryotes and from 0.37 to 0.57 for fungi. The first two axes of the principal coordinates analysis (PCoA) explained 60.2% of the variation in the prokaryotic profiles (44.1% and 16.2%) and 60.6% in the fungal profiles (44.5% and 16.1%) (Figure 2). In the prokaryotic ordination, all five rainfed profiles were located at negative values of the first axis, whereas four of the five irrigated profiles were at positive values; in the fungal ordination, the five rainfed profiles were at higher values of the second axis than the irrigated ones (Figure 2). Profiles did not cluster by season in either ordination.

For prokaryotes, the two-factor PERMANOVA attributed 23.7% of the variation to management (*R*^2^ = 0.237; pseudo-*F* = 2.42; *p* = 0.048), 12.1% to season (*p* = 0.245) and 5.3% to their interaction (*p* = 0.846) (Table 2). Management labels were permuted only within campaigns, which respects the paired sampling dates, and the exact *p*-value was 0.125 (smallest attainable value, 0.031). Dispersion did not differ significantly between managements (PERMDISP, *F* = 6.18, *p* = 0.121), although irrigated profiles were more dispersed (mean distance to the centroid 0.191 vs. 0.117); differences in dispersion may therefore contribute to the management term. Across taxonomic ranks, the management term for prokaryotes was significant at phylum, class, order, genus and species level (*R*^2^ between 0.127 at order level and 0.302 at phylum level; Table S4).

For fungi, the management explained 15.1% of the variation (*R*^2^ = 0.151; pseudo-*F* = 1.26; *p* = 0.269), season 8.5% (*p* = 0.641) and the interaction 4.4% (*p* = 0.982), with no difference in dispersion between managements (*p* = 0.707). The restricted permutation test gave *p* = 0.062. Thus, although fungal profiles differed between managements within every campaign (Bray–Curtis 0.37–0.57), the campaign-to-campaign variation was of similar magnitude, and a management effect could not be distinguished with ten profiles. As a sensitivity analysis, the spring 2026 campaign was excluded (*n* = 8 profiles per marker). The management term then became significant for both fungi (*R*^2^ = 0.276, *p* = 0.007) and prokaryotes (*R*^2^ = 0.436, *p* = 0.003), whereas season remained non-significant (fungi *p* = 0.229; prokaryotes *p* = 0.296) (Table S4). The spring 2026 fungal profiles had incomplete taxonomic assignment; the conclusion about a management association is therefore sensitive to the inclusion of this campaign and should be interpreted cautiously. The limited number of profiles, the unbalanced seasonal sampling (six spring and four autumn profiles) and the absence of autumn 2026 samples further limit the power to detect seasonal or interaction effects.

### 2.4. Relative Taxonomic Composition

**Figure 3:**
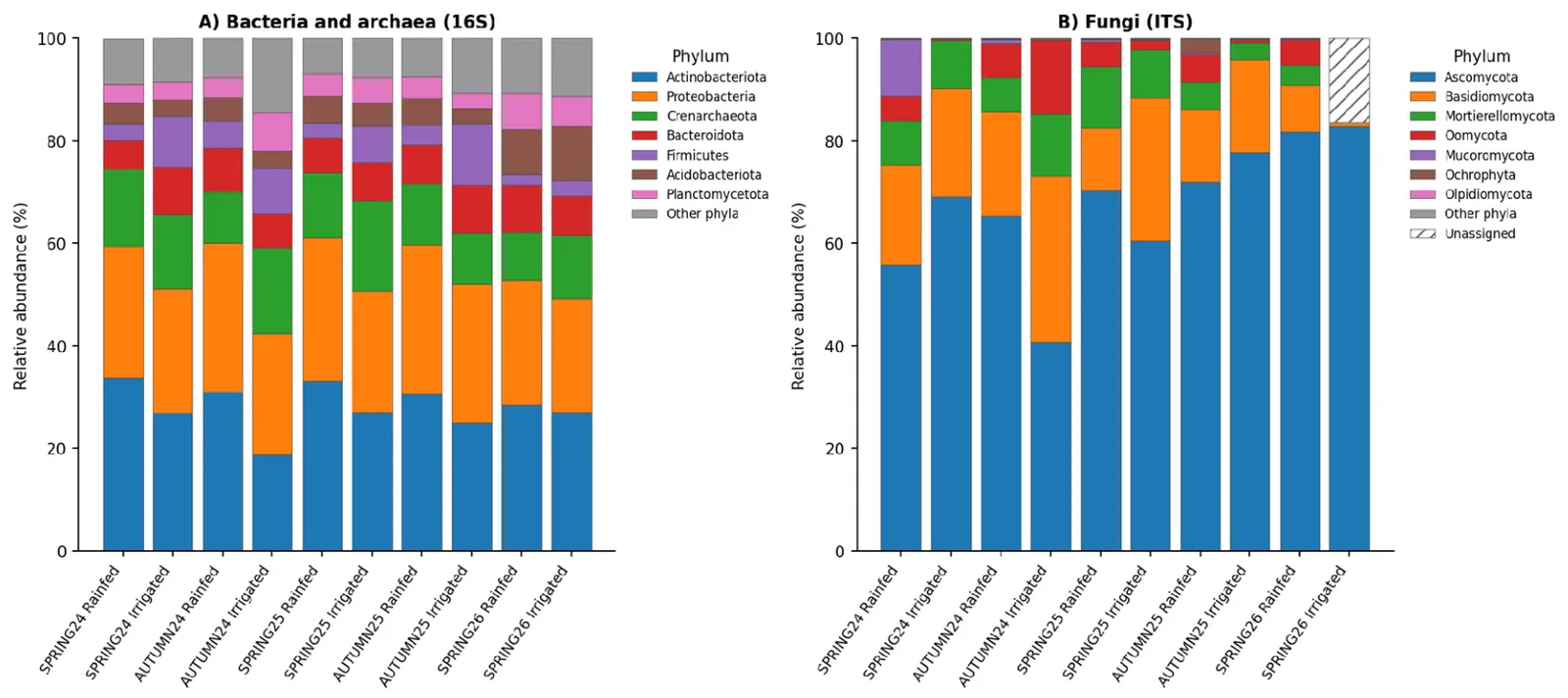
**Relative taxonomic composition at the phylum level.** Stacked bars show the percentage composition of (**A**) the seven most abundant prokaryotic phyla (16S rRNA gene; Crenarchaeota are Archaea) and (**B**) the seven most abundant fungal phyla (ITS1) in rainfed and irrigated vineyards in the five sampling campaigns (spring 2024 to spring 2026; *n* = 10 profiles per marker). Remaining phyla are grouped as ‘Other phyla’. The hatched bar in panel B indicates reads not assigned at phylum level in the irrigated spring 2026 profile.

**Phylum level.** The 16S profiles were dominated by Actinobacteriota, Proteobacteria, Crenarchaeota (Archaea), Bacteroidota and Acidobacteriota (Figure 3A). Archaea accounted for 9.3–17.6% of the 16S profiles (mean 11.8% in rainfed and 14.4% in irrigated vineyards), almost entirely Crenarchaeota; the 16S dataset is therefore referred to as prokaryotic throughout. Actinobacteriota (31.3% (28.4–33.7) in rainfed vs. 24.9% (18.7–27.0)) in irrigated vineyards; mean and range across campaigns) and Proteobacteria (27.3% (24.4–29.3) vs. 24.2% (22.2–27.0)) had higher relative abundance in rainfed vineyards in all five campaigns, whereas Firmicutes were more abundant in irrigated vineyards in all five (3.5% (2.0–5.3) in rainfed vs. 8.2% (2.9–12.0) in irrigated). Acidobacteriota were higher in rainfed vineyards in four campaigns (5.5% (4.1–8.8) vs. 5.0% (3.1–10.7)), while Crenarchaeota (11.8% (9.3–14.9) vs. 14.3% (9.9–17.6)), Bacteroidota (7.6% (5.7–9.3) vs. 8.1% (6.5–9.4)) and Planctomycetota (4.6% (3.6–7.2) vs. 4.9% (2.8–7.5)) showed no consistent pattern.

The ITS1 profiles were dominated by Ascomycota, followed by Basidiomycota and Mortierellomycota (Figure 3B). Ascomycota did not show a consistent pattern between managements: the relative abundance was 69.0% (55.8–81.8) in rainfed and 66.2% (40.7–82.9) in irrigated vineyards, and was higher in irrigated vineyards in three of the five campaigns. Basidiomycota (15.1% (9.2–20.3) in rainfed vs. 20.1% (0.7–32.4) in irrigated) and Mortierellomycota (7.3% (3.9–11.9) vs. 6.8% (0.0–12.0)) also varied widely among campaigns. In the irrigated spring 2026 profile, only 83.6% of the reads were assigned at phylum level, and this profile is shown with an ‘unassigned’ category in Figure 3B. These comparisons are descriptive; no differential abundance test was performed because of the small number of profiles per management. The tendency toward higher fungal dominance in irrigated vineyards (Section 2.2) was therefore not explained at the phylum level, but may reflect a few genera, notably *Solicoccozyma* and *Fusarium* (Table 3).

**Taxa related to nutrient cycling and plant health.** To relate the taxonomic data to potential ecosystem functions, we summed the relative abundance of selected genera that include taxa associated with nitrogen cycling, plant growth promotion or pathogenicity (Table 3). These groups are inferred from taxonomy alone and represent hypotheses about potential function, not measured activity. Ammonia-oxidizing archaea (*Nitrososphaera* and *Nitrosocosmicus*) were the most abundant group (mean 11.8% in rainfed and 14.2% in irrigated vineyards), and nitrifying bacteria represented less than 1% of the reads in both managements. *Bacillus* was more abundant in irrigated vineyards in all five campaigns (1.4% vs. 2.6%). Among fungi, *Fusarium* was more abundant in irrigated vineyards (1.9% vs. 4.4%; higher in four of five campaigns) and *Trichoderma* in rainfed vineyards (0.49% vs. 0.08%; higher in all five campaigns), and *Solicoccozyma* reached 14.9% in irrigated vs. 5.4% in rainfed vineyards on average. *Cadophora* was detected at very low relative abundance (≤1.2%) and was not detected in two irrigated profiles.

**Arbuscular mycorrhizal fungi.** Glomeromycota, the phylum that contains most arbuscular mycorrhizal fungi (AMF), was detected only at trace levels in rainfed vineyards (0.005–0.040% of the ITS1 reads in the five rainfed profiles) and was not detected in any irrigated profile (Table S5). Given the known under-representation of Glomeromycota in ITS1 amplicon data [23,24], these results are interpreted as near non-detection under the selected assay and not as evidence of absence.

### 2.5. Bacteria-to-Fungi Ratio

**Figure 4:**
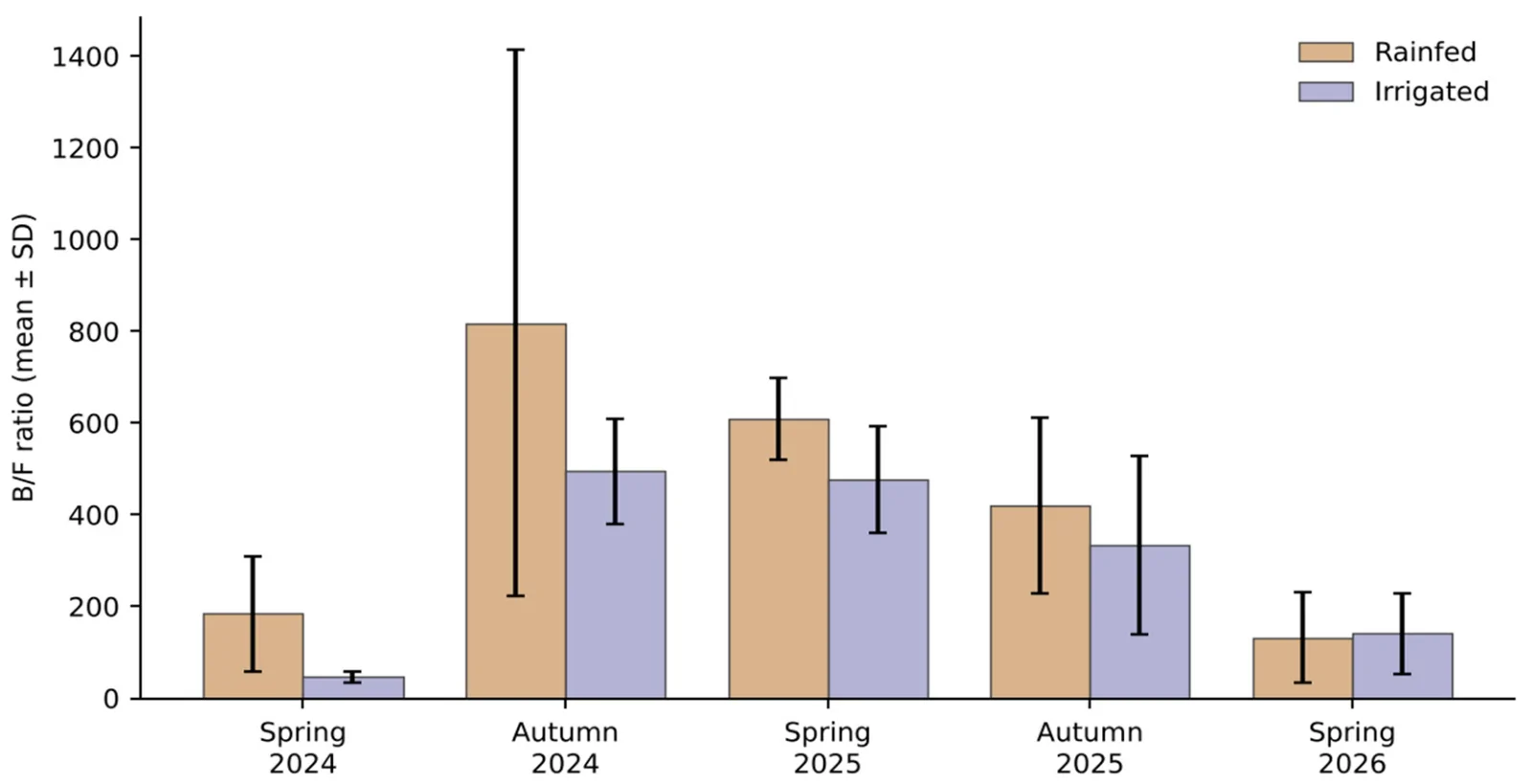
**Bacteria-to-fungi (B/F) ratio by campaign and management.** Bars show the mean and error bars the standard deviation in rainfed (tan) and irrigated (purple) vineyards. The ratio is a sequencing-derived index and was not tested statistically.

The bacteria-to-fungi (B/F) ratio, a sequencing-derived index (see Methods 4.5), ranged from 46.0 to 494.6 in irrigated and from 131.7 to 817.9 in rainfed vineyards across campaigns (Table 4; Figure 4). It was higher in rainfed than in irrigated vineyards in four of the five campaigns (in spring 2026 the values were similar: 131.7 vs. 140.6) and tended to be higher in autumn (mean 517; range 333–818) than in spring (mean 265; range 46–609). However, the standard deviations among replicate samples were large and the ranges overlapped between managements. Given the small number of observations, the undefined pairing structure of the design and the nature of the index (see Discussion), the B/F values are reported descriptively and no inferential tests were applied.

Overall, the results show that fungal richness, diversity and evenness were consistently lower in irrigated than in rainfed vineyards, that prokaryotic alpha diversity did not differ consistently, and that associations between management and community composition were modest and sensitive to the campaigns included. These patterns must be read together with the confounding factors and limited replication discussed below.

## 3. Discussion

This observational study compared the rhizosphere communities of rainfed and irrigated ‘Pardina’ vineyards over five campaigns. The clearest and most consistent signal was a lower richness, diversity and evenness of fungi, with a tendency toward higher dominance of a few genera (not significant after correction), in irrigated vineyards. Prokaryotic alpha diversity did not differ consistently, and the association between management and community composition was modest for prokaryotes, undetected for fungi when all campaigns were included, and sensitive to the inclusion of spring 2026. The comparison is confounded with planting density (<3000 vs. >3000 vines ha^−1^) and with soil properties (pH, available phosphorus and organic carbon; Table S1), and individual vineyards are not replicated within each management, so individual soil samples cannot be treated as independent replicates of the treatment [25]. The results therefore describe associations between two management systems as they occur in Tierra de Barros, and they should not be interpreted as effects of irrigation itself.

Lower fungal diversity under irrigation is in line with reports that irrigation and drought modify the fungal communities of vineyard soils and rhizosphere [23,24], and that fungi respond to water management in other Mediterranean and arid agroecosystems [26,27]. A possible but untested explanation is that the more stable moisture of irrigated soils reduces microhabitat heterogeneity and favors fast-growing, resource-exploiting taxa [12,28,29,30,31,32]. In our data, genera such as *Solicoccozyma* and *Fusarium* were more abundant in irrigated profiles (Table 3), which agrees with a more dominated community. However, the same pattern could arise from differences in planting density or from the soil contrasts we observed (pH 8.14 in rainfed vs. 7.65 in irrigated soils; available P 21 vs. 48 ppm; organic carbon 0.74 vs. 0.99%), which are known determinants of fungal community structure [5,13,14]. The lower pH, carbonate content and exchangeable Ca of irrigated soils (pH 7.65 vs. 8.14; carbonates 1.82 vs. 3.58%; exchangeable Ca 17.96 vs. 21.41 meq 100 g^−1^; Table S1) are consistent with the carbonate chemistry of Calcaric Cambisols. In calcareous soils, pH and dissolved Ca^2+^ are controlled by the CaCO_3_–CO_2_–H_2_O equilibrium, and a higher soil CO_2_ partial pressure, as expected from greater root and microbial respiration in moister soils, lowers pH and increases calcite solubility [33]. Irrigation has also been associated with the depletion and redistribution of carbonates in the tilled layer of a calcareous soil [34], and the irrigation water used here, collected rainwater, is unlikely to replenish Ca^2+^ or bicarbonate. Soil pH and Ca concentration are among the strongest edaphic predictors of fungal richness at the global scale [35], so these changes might contribute to the lower fungal diversity under irrigation. However, fungal diversity is usually much less sensitive to pH than bacterial diversity: along a pH 4.0–8.3 gradient, bacterial richness roughly doubled, whereas fungal richness showed only a weak non-significant trend [36]. The difference observed here (≈0.5 pH units within the alkaline range) is smaller than the within-management standard deviations, and prokaryotic alpha diversity, which would be expected to respond more strongly to pH, did not differ consistently between managements. The pH and Ca contrasts are therefore unlikely to be the main driver of the lower fungal diversity; soil moisture itself and the other soil and management contrasts are at least plausible explanations, particularly because fungal communities are generally more resistant to drought than bacterial communities [37], so rainfed conditions need not depress fungal diversity. Because the soil data are available only as means by management and season, we could not test these associations, and disentangling them requires replicated plots with plot-level soil measurements.

For prokaryotes, the association with management was weaker (*R*^2^ = 0.237) and not robust to permutations restricted within campaigns. Irrigated profiles were also more dispersed, so part of the management term may reflect heterogeneity among campaigns and not a shift in location. The tendency toward higher Actinobacteriota and Proteobacteria and lower Firmicutes in rainfed vineyards is descriptive. Differences in prokaryotic composition between irrigation managements have been reported in vineyards [38] and other crops [39], and our results are compatible with them but do not add independent support. The 16S dataset included Archaea, mainly Crenarchaeota, which represented 9–18% of the profiles and are largely ammonia oxidizers; we therefore refer to the prokaryotic community and do not describe it as bacterial.

Regarding practical relevance, the taxa linked to nitrogen cycling (ammonia-oxidizing archaea, nitrifying bacteria and Rhizobiales) were present in both managements, with ammonia-oxidizing archaea and nitrifying bacteria at similar or slightly higher relative abundance in irrigated soils and Rhizobiales slightly lower (Table 3). This does not suggest an obvious loss of nitrifier potential under irrigation. The higher relative abundance of *Fusarium* and the lower abundance of *Trichoderma* in irrigated soils could point to a different balance between potentially pathogenic and biocontrol-associated fungi, but these genera include both beneficial and harmful species, and our data do not resolve species or function. Taxonomic composition does not demonstrate function, and the relationship between diversity, composition and soil processes depends on the soil environment and on the microbial assemblage [40,41]. No enzymatic activity, nitrification or mineralization rates, nutrient availability linked to the microbiome, disease incidence, vine performance or mycorrhizal colonization were measured. Statements about soil health or multifunctionality cannot therefore be derived from this study; they are hypotheses to be tested.

The season and the management × season interaction were not significant. With ten profiles, six spring and four autumn campaigns, no autumn 2026 sampling and year partly confounded with season, the power to detect seasonal effects is low, and a non-significant result is not evidence that season is unimportant. Root exudates can structure rhizosphere communities and might buffer temporal variation [39,42,43], but that mechanism cannot be inferred from the lack of a significant seasonal effect and was not tested here.

Glomeromycota was detected at trace levels in rainfed vineyards and not in irrigated vineyards. This pattern should not be interpreted as an absence of AMF, because ITS1 primers under-represent Glomeromycota relative to LSU or SSU markers [44,45], and root colonization, spore counts and positive controls were not available. We therefore make no inference about AMF status under either management, and we do not link AMF detection to the occurrence of *Cadophora* or to dysbiosis.

The B/F ratio was calculated from independent 16S and ITS libraries. The two markers differ in rRNA gene copy number among taxa [46,47], in primer coverage and amplification efficiency, and in library composition, so the ratio is not a measure of relative biomass or abundance of bacteria and fungi [24,48]. We report it descriptively as an exploratory index. It tended to be higher in rainfed vineyards (four of five campaigns) and in autumn, but the variability among replicates was large. Robust B/F comparisons would require quantitative methods such as qPCR, phospholipid fatty acid analysis or microbial biomass measurements.

Several limitations should be considered. First, the design is observational, with limited plot-level replication within each management and a partial confounding of management with planting density and soil properties. Second, the community data are relative abundances (compositional) [49] aggregated into ten profiles per marker, and the soil data are available only as means; therefore, formal tests of the association between soil variables and community structure were not possible. Finally, amplicon data provide taxonomic information only.

Future work should use replicated plots for each management, quantify irrigation and soil moisture, measure plot- and date-specific soil properties, and combine amplicon sequencing with functional measurements (enzyme activities, nitrification potential, qPCR and mycorrhizal colonization). Controlled deficit irrigation and the use of cover crops are management options that can be evaluated in such experiments [24,44]; our data do not show that they would preserve microbial diversity or soil multifunctionality.

## 4. Materials and Methods

### 4.1. Study Area

This study was conducted in a 30 ha located in the Tierra de Barros region (Badajoz, Extremadura, Spain; 38°44′–38°52′ N, 6°28′–6°44′ W). The area has an arid/semi-arid Mediterranean climate with marked seasonal variations in precipitation and hot, dry summers. The average annual temperature is approximately 16 °C (with average lows of ≈11 °C and highs of ≈23 °C), average annual sunshine hours of 2800–3000 h, and cumulative reference potential evapotranspiration (ETo) of ≈1250 mm·year^−1^ [50].

Geomorphologically, the physiography consists of flat or gently undulating terrain [51]. Lithologically, Tertiary detrital materials predominate—primarily from the Miocene and Pliocene—composed of sandstones, conglomerates, gravels, sands, silts, and red clays, as well as Quaternary slope deposits containing caliches and calcareous crusts [52]. The soils developed on this parent material are representative of the Mediterranean viticultural agrosystems of the Iberian Peninsula, classified predominantly as Calcaric Cambisols and, to a lesser extent, Chromic Luvisols [53,54]. These soils are characterized by a subsurface B horizon that is cambic, argillic, or calcareous; a variable carbonate content; loamy to clayey textures; a neutral to alkaline pH; and low organic matter content [52,55].

In Tierra de Barros, the cultivars ‘Pardina’ and ‘Cayetana Blanca’ are grown, ‘Pardina’ being the most widespread. General agronomic management included shallow tillage, no inter-row plant cover, mechanized harvesting, synthetic mineral fertilization (N-P-K), and conventional application of agrochemicals (herbicides, insecticides, and fungicides), in both rainfed and irrigated vineyards. Planting density differed, with <3000 vines·ha^−1^ recorded under rainfed conditions and >3000 vines·ha^−1^ under irrigated conditions.

Our study, an observational comparison, was carried out in vineyards of the cultivar ‘Pardina’ under both rainfed and irrigated conditions and subjected to the typical management of Tierra de Barros. Three rainfed plots and three irrigated plots were selected within an area of 30 ha. Management was not an experimental treatment. Stratified random sampling of soil took place near the base of the vine: 6 during spring of 2024, (3 from rainfed and 3 from irrigated vineyards) and 6 during autumn of 2024, (3 from rainfed and 3 from irrigated vineyards). A total of 30 samples of soil, from the base of vine, were collected: 18 in the spring (9 from rainfed and 9 from irrigated vineyards) and 12 in the autumn (6 from rainfed and 6 from irrigated vineyards), consecutive campaigns (2024–2026). On irrigated plots, drip irrigation is applied at the base of the vine; irrigation frequency is 2.6–3.6 mm/ha; irrigation water comes from artificial reservoirs that collect rainwater, which is used throughout the growing season. Three rainfed plots and three irrigated plots were selected within an area of 30 ha. Plot-level characteristics are listed in Table S6.

### 4.2. Rhizosphere Soil Sampling and Preservation

The bulk soil samples were collected in the 0–30 cm depth layer near the base of the vine. The rhizosphere samples consisted of the soil fraction closely adhering to the active root system in the 0–20 cm depth layer, extracted near the trunk following the standard methodology for microbiome studies in vineyards [11,56,57]. Each campaign × management combination was represented by three samples (3 campaigns × 2 managements × 3 samples = 18 in spring; 2 campaigns × 2 managements × 3 samples = 12 in autumn). The samples from each campaign and treatment group were combined into a single community profile, and the mean value was calculated after sequencing, so that the community dataset comprises ten profiles per marker (five campaigns × two managements), and the unit of analysis is the campaign × management profile. The same vines were sampled in all campaigns. Spring samples were collected during the second week of April, and autumn samples during the first week of November; each sample consisted of 4 vines. The distance from the trunk at which samples were collected was 50 cm for soil bulk samples and 10 cm for soil rhizosphere samples. From each sampled vine, 4 roots were collected, resulting in a total of approximately 100 g of soil per vine. After extraction, the material was handled under aseptic conditions and homogenized to minimize intrasample variability by mixing and then taking a 100 g sample [17,58,59]. The homogenized samples were immediately frozen and stored at −80 °C until molecular processing to preserve genomic DNA integrity and prevent alterations in community composition [60]. In all the plots studied, the usual agronomic practices involving the application of agrochemicals were carried out at the appropriate times; in the irrigated plots, irrigation took place from May through August, coinciding with the period of greatest water deficit. The samples of bulk soils were air-dried, sieved to 2 mm and ground for subsequent analysis. Soil physicochemical properties (Table S1) were determined as follows: Particle size distribution was determined using the Bouyoucos method, after the removal of organic matter with H_2_O_2_ and carbonates with HCl [61]. Soil pH was measured potentiometrically using a 1:1 soil-to-water ratio (weight/volume). Electrical conductivity (EC) was determined in saturated soil paste. Available phosphorus was determined by the Olsen method [62]. Cation exchange capacity (CEC) and exchangeable bases were determined using a 1 M NH_4_OAc solution buffered at pH 7. Potassium (K^+^) and sodium (Na^+^) were quantified by flame photometry, and calcium (Ca^2+^) and magnesium (Mg^2+^) by atomic absorption spectrometry (AAS) [62]. Soil organic carbon (SOC) was determined according to the Walkley–Black procedure, and total nitrogen (TN) was obtained at a rate of 5% of organic matter [63]. Calcium carbonate content was determined volumetrically using a Bernard calcimeter [62].

### 4.3. DNA Extraction, PCR Amplification, and Sequencing

Total genomic DNA was isolated from rhizosphere soil using the DNeasy PowerLyzer PowerSoil kit (Qiagen, Hilden, Germany) according to the manufacturer’s specifications for the BeCrop^®^ platform (Biome Makers Inc., Valladolid, Spain). The characterization of bacterial and fungal communities was performed by PCR amplification of the V4 hypervariable region of the 16S rRNA gene and the internal transcribed spacer 1 (ITS1) region, respectively. The generated amplicons were purified using KAPA Pure Beads magnetic microspheres (Roche, Basel, Switzerland), and the quality of the amplification product was verified by agarose gel electrophoresis.

### 4.4. Bioinformatics Processing and Taxonomic Assignment

Taxonomic assignment was performed by aligning the representative sequences against the curated databases SILVA v138 for the 16S rRNA gene and UNITE v8.3 for the ITS region [64]. The analyses reported here were run on the relative abundance tables (percentage of assigned reads per taxon) of each campaign × management profile, which are independent of library size [65].

The samples were analyzed by Biome Makers Spain, S.L., and the results are available in an open and free atlas of soil microorganisms at a global scale (BeOpen; https://beopen.earth/; accessed on 26 October 2026). BeOpen makes it possible to consult the distribution of soil bacteria and fungi, showing in which 50 km^2^ cells each genus and species has been detected, based on more than 150,000 samples analyzed with the same laboratory protocol and homogeneous bioinformatic processing.

### 4.5. Ecological and Statistical Analysis

Analyses were carried out at genus level, the rank for which the tables of all twenty profiles (two markers × two managements × five campaigns) were internally consistent. Where the genus-level percentages did not sum to 100% (fungal profiles of spring 2026, ≈83%), abundances were renormalized to 100%, and the analyses were repeated without that campaign as a sensitivity check (Table S4).

Alpha diversity was described with observed genus richness (*S*); the Shannon diversity index (*H*′, natural logarithm), which combines richness and evenness and is not itself an evenness index; Pielou’s evenness (*J* = *H*′/ln *S*); Berger–Parker dominance (relative abundance of the most abundant genus); and the Gini coefficient of the abundance distribution. The Chao1 estimator was not used because it requires read counts, which are not available in the relative abundance tables. Differences between managements were estimated as the mean paired difference across the five campaigns (irrigated/rainfed; metric ∼ campaign + management) with 95% confidence intervals from the *t* distribution (df = 4), paired *t* tests and exact Wilcoxon signed-rank tests; *p*-values were adjusted with the Holm procedure across the five metrics within each marker. Campaign was used as a blocking factor because both managements were sampled in the same campaign; however, vineyards are not replicated within management, so these tests describe the consistency of the contrast across campaigns and do not establish treatment effects [25].

Beta diversity was assessed with Bray–Curtis dissimilarities calculated from relative abundances and visualized by principal coordinates analysis (PCoA). The effects of management, season and their interaction were tested with a two-factor permutational multivariate analysis of variance (PERMANOVA) [66] with sequential sums of squares and 9999 permutations of the ten profiles. Because both managements were sampled in each campaign, the management term was also tested by permuting management labels only within campaigns (all 2^5^ = 32 label swaps; the smallest attainable *p*-value is 0.031). Homogeneity of multivariate dispersion (PERMDISP) [67] was tested for management and season with 9999 permutations. Sensitivity to taxonomic rank was examined by repeating the two-factor PERMANOVA without the interaction term (999 permutations) at phylum, class, order, genus and (for prokaryotes) species level, and sensitivity to the spring 2026 campaign by excluding it. Relative abundance data are compositional [49]; comparisons of individual taxa are therefore descriptive (means, ranges and numbers of campaigns), and no differential abundance test was performed.

The B/F ratio was provided as units per gram of soil of bacteria versus units per gram of soil of fungi. It is a sequencing-derived exploratory index and was analyzed descriptively. All analyses and figures were produced in Python (NumPy, SciPy, pandas, Matplotlib); PERMANOVA, PERMDISP and PCoA were implemented following Anderson [66,67]. A significance level of α = 0.05 was used.

## 5. Conclusions

In this observational comparison of rainfed and irrigated ‘Pardina’ vineyards, fungal communities in irrigated vineyards showed consistently lower richness, Shannon diversity and evenness than those in rainfed vineyards in all five campaigns, with a tendency toward a higher dominance of a few genera, whereas prokaryotic alpha diversity did not differ consistently.

Management was modestly associated with prokaryotic community composition (*R*^2^ = 0.237, *p* = 0.048) and not detectably with fungal composition (*R*^2^ = 0.151, *p* = 0.269) when all campaigns were analyzed; both results were sensitive to the inclusion of spring 2026, and season and its interaction with management was not significant. These findings must be interpreted considering the observational design, the confounding of management with planting density and soil properties, the limited plot-level replication, the unbalanced seasonal sampling and the absence of autumn 2026 samples.

The bacteria-to-fungi ratio is a sequencing-derived index that was reported descriptively and does not measure relative biomass. No soil function, plant health or mycorrhizal colonization was measured, so the implications for soil health, multifunctionality or deficit irrigation remain hypotheses. They should be tested in replicated experiments with quantified irrigation, plot-level soil properties and functional measurements.

## Figures and Tables

**Table 1:** Alpha diversity (genus level) of the prokaryotic and fungal rhizosphere communities in rainfed and irrigated vineyards. Values are medians (interquartile range) of the five campaign profiles per management. Δ is the mean paired difference (irrigated/rainfed) with its 95% confidence interval (*t* distribution, df = 4). *p*, paired *t* test; *p*_Holm_, Holm-adjusted *p* within each marker (five metrics). Full statistics, including exact Wilcoxon tests, are given in Table S2. The two managements were sampled in the same campaigns, but vineyards are not replicated within managements; the *p*-values therefore describe the consistency of the contrast across campaigns.

Metric	Rainfed, Median (IQR)	Irrigated, Median (IQR)	Δ (95% CI)	*p*	*p*_Holm_
**Prokaryotes (16S rRNA gene)**					
Observed genus richness (*S*)	488 (442–554)	414 (398–453)	−32 (−137 to 73)	0.446	0.892
Shannon index (*H*′)	4.51 (4.38–4.52)	4.47 (4.44–4.57)	0.05 (−0.15 to 0.26)	0.489	0.892
Pielou’s evenness (*J*)	0.728 (0.714–0.730)	0.738 (0.737–0.752)	0.017 (0.004 to 0.030)	0.020	0.102
Berger–Parker dominance (%)	7.6 (7.1–7.7)	9.0 (8.3–10.1)	1.3 (−1.4 to 4.1)	0.241	0.722
Gini coefficient	0.848 (0.839–0.849)	0.812 (0.789–0.837)	−0.026 (−0.048 to −0.004)	0.029	0.118
**Fungi (ITS1)**					
Observed genus richness (*S*)	297 (293–307)	180 (135–184)	−130 (−163 to −96)	<0.001	0.002
Shannon index (*H*′)	3.84 (3.80–3.86)	3.05 (2.92–3.19)	−0.73 (−1.09 to −0.38)	0.005	0.019
Pielou’s evenness (*J*)	0.671 (0.669–0.694)	0.615 (0.595–0.617)	−0.063 (−0.100 to −0.026)	0.009	0.027
Berger–Parker dominance (%)	11.9 (10.4–12.3)	19.3 (18.7–21.4)	8.6 (0.2 to 17.1)	0.047	0.095
Gini coefficient	0.858 (0.835–0.873)	0.880 (0.869–0.885)	0.015 (−0.010 to 0.040)	0.166	0.166

**Table 2:** Two-factor PERMANOVA on Bray–Curtis dissimilarities (genus level) of prokaryotic and fungal community profiles. Sequential (type I) sums of squares; 9999 permutations of the ten profiles. The full model was Bray–Curtis ∼ management + season + management × season.

Term	df	SS	*R* ^2^	Pseudo-*F*	*p*
**Prokaryotes (16S rRNA gene)**					
Management	1	0.0833	0.237	2.42	0.048
Season	1	0.0426	0.121	1.24	0.245
Management × Season	1	0.0187	0.053	0.54	0.846
Residual	6	0.2064	0.588		
Total	9	0.3510	1.000		
**Fungi (ITS1)**					
Management	1	0.2147	0.151	1.26	0.269
Season	1	0.1211	0.085	0.71	0.641
Management × Season	1	0.0631	0.044	0.37	0.982
Residual	6	1.0244	0.720		
Total	9	1.4232	1.000		

**Table 3:** Relative abundance (%) of selected genera and genus groups in rainfed and irrigated vineyards. Values are the mean (minimum/maximum) of the five campaign profiles. Functional labels are inferred from taxonomy and were not measured.

Genus or Group	Rainfed, Mean (Min–Max)	Irrigated, Mean (Min–Max)	Campaigns Irrigated > Rainfed
**Prokaryotes**			
Ammonia-oxidizing archaea (*Nitrososphaera* + *Nitrosocosmicus*)	11.78 (9.29–14.94)	14.18 (9.91–17.58)	3/5
Nitrifying bacteria (*Nitrospira*, *Nitrosospira*, *Nitrosomonas*, *Nitrobacter*)	0.58 (0.41–0.80)	0.77 (0.58–1.00)	4/5
Rhizobiales (*Bradyrhizobium* + *Mesorhizobium*)	0.61 (0.48–0.83)	0.47 (0.32–0.61)	1/5
*Bacillus*	1.40 (0.77–2.36)	2.61 (0.95–3.66)	5/5
**Fungi**			
*Mortierella* (saprotroph)	6.54 (0.00–11.89)	6.81 (0.00–11.98)	2/5
*Trichoderma*	0.49 (0.15–1.08)	0.08 (0.05–0.14)	0/5
*Fusarium*	1.91 (0.83–3.13)	4.41 (1.21–7.42)	4/5
*Cadophora*	0.10 (0.00–0.35)	0.27 (0.00–1.17)	3/5
*Solicoccozyma*	5.39 (0.00–9.62)	14.91 (0.00–27.73)	3/5

**Table 4:** Bacteria-to-fungi (B/F) ratio by campaign and management (mean ± SD). The ratio is a sequencing-derived index and not a measure of relative biomass.

Campaign	Rainfed	Irrigated
Spring 2024	183.8 ± 125.3	46.0 ± 12.8
Autumn 2024	817.9 ± 595.9	494.6 ± 115.9
Spring 2025	608.5 ± 88.7	476.5 ± 116.7
Autumn 2025	421.2 ± 191.7	332.8 ± 194.6
Spring 2026	131.7 ± 98.0	140.6 ± 87.3

## Data Availability

Soil physicochemical data supporting this study are provided as Supplementary Materials. The genus-level relative abundance data are available at https://beopen.earth/.

## Supplementary Materials

The following supporting information can be downloaded at: https://www.mdpi.com/article/10.3390/plants15213468/s1. Table S1, soil physicochemical properties by management and season; Table S2, full alpha diversity statistics; Table S3, PERMANOVA with restricted permutations and PERMDISP; Table S4, sensitivity analyses (taxonomic rank and exclusion of spring 2026); Table S5, phylum-level composition and Glomeromycota; Table S6, vineyard characteristics; analysis code and genus-level relative abundance tables.
