# Supplementary material for: Effects of Irrigation Regime and Seasonal Dynamics on the Rhizosphere Microbiome on Calcaric Cambisols in Western Iberian Vineyards: An Observational Study Across Seasons
Source: Plants (Basel). 2026 Nov 6;15(21):3468. doi: 10.3390/plants15213468 (PMC13765682; doi:10.3390/plants15213468)
Supplement: Supplementary file 1 [file plants-15-03468-s001.zip › plants-4628157-supplementary.pdf]

Supplementary Material

Rhizosphere Microbiome of Rainfed and Irrigated Vineyards on Calcaric Cambisols in Western Iberia: An Observational Study Across Seasons.

Fernández-Pozo and Rodríguez-González

All community analyses use genus-level relative-abundance tables of ten campaign × management profiles per marker (five campaigns × two managements).

Table S1. Soil physicochemical properties

Mean ± standard deviation by management and by season, as supplied. n= 12

Property	Rainfed	Irrigated	Spring	Autumn
Sand (%)	63.81 ± 4.89	65.00 ± 6.94	64.48 ± 5.77	63.66 ± 6.38
Clay (%)	16.37 ± 3.17	15.77 ± 7.79	15.86 ± 6.08	16.80 ± 5.99
Silt (%)	19.82 ± 2.83	19.23 ± 7.00	19.66 ± 4.76	19.55 ± 5.85
Bulk density (Mg m^{-3})	1.68 ± 0.23	1.54 ± 0.17	1.62 ± 0.23	1.66 ± 0.25
pH	8.14 ± 0.77	7.65 ± 0.86	7.82 ± 0.91	7.96 ± 0.99
Electrical conductivity (dS m^{-1})	0.20 ± 0.08	0.33 ± 0.27	0.32 ± 0.26	0.200 ± 0.13
Carbonates (%)	3.58 ± 5.03	1.82 ± 1.07	2.67 ± 4.12	2.73 ± 4.02
Available P (ppm)	21.29 ± 5.84	48.05 ± 25.60	35.91 ± 30.52	33.44 ± 36.16
CEC (meq 100 g^{-1})	16.16 ± 6.40	14.74 ± 3.33	15.54 ± 4.94	15.36 ± 5.66
Exchangeable Na (meq 100 g^{-1})	0.22 ± 0.32	0.28 ± 0.14	0.24± 0.16	0.27 ± 0.33
Exchangeable K (meq 100 g^{-1})	0.64 ± 0.28	0.87 ± 0.32	0.78 ± 0.41	0.73 ± 0.35
Exchangeable Ca (meq 100 g^{-1})	21.41 ± 12.15	17.96 ± 10.22	20.23 ± 12.35	19.14 ± 11.08
Exchangeable Mg (meq 100 g^{-1})	1.71 ± 0.79	2.46 ± 0.95	2.19 ± 1.26	1.98 ± 1.02
Soil organic carbon (%)	0.74 ± 0.26	0.99 ± 0.33	0.96 ± 0.38	0.77 ± 0.30
Total N (%)	0.055 ± 0.020	0.07 ± 0.02	0.07 ± 0.03	0.06 ± 0.02

Table S2. Alpha diversity: full paired statistics

Metrics at genus level; n = 5 campaign profiles per management. Δ = irrigated − rainfed (mean of paired differences); t test with df = 4; exact Wilcoxon signed-rank test (smallest attainable two-sided p = 0.0625); Holm adjustment across five metrics within each marker; d_z, Cohen's paired effect size.

Marker	Metric	Rainfed median (IQR)	Irrigated median (IQR)	Δ (95% CI)	Campaigns irrigated < rainfed	t	p (t)	pHolm	p (Wilcoxon)	d_z
Prokaryotes	Observed genus richness (S)	488 (442–554)	414 (398–453)	−32 (−137 to 73)	3	-0.84	446	892	0.6250	-0.38
Prokaryotes	Shannon index (H′)	4.51 (4.38–4.52)	4.47 (4.44–4.57)	0.05 (−0.15 to 0.26)	2	0.76	489	892	0.6250	0.34
Prokaryotes	Pielou's evenness (J)	0.728 (0.714–0.730)	0.738 (0.737–0.752)	0.017 (0.004 to 0.030)	0	3.72	20	102	0.0625	1.66
Prokaryotes	Berger–Parker dominance (%)	7.6 (7.1–7.7)	9.0 (8.3–10.1)	1.3 (−1.4 to 4.1)	1	1.38	241	722	0.4375	0.62
Prokaryotes	Gini coefficient	0.848 (0.839–0.849)	0.812 (0.789–0.837)	−0.026 (−0.048 to −0.004)	5	-3.32	29	118	0.0625	-1.48
Fungi	Observed genus richness (S)	297 (293–307)	180 (135–184)	−130 (−163 to −96)	5	-10.85	<0.001	2	0.0625	-4.85
Fungi	Shannon index (H′)	3.84 (3.80–3.86)	3.05 (2.92–3.19)	−0.73 (−1.09 to −0.38)	5	-5.71	5	19	0.0625	-2.55
Fungi	Pielou's evenness (J)	0.671 (0.669–0.694)	0.615 (0.595–0.617)	−0.063 (−0.100 to −0.026)	5	-4.74	9	27	0.0625	-2.12
Fungi	Berger–Parker dominance (%)	11.9 (10.4–12.3)	19.3 (18.7–21.4)	8.6 (0.2 to 17.1)	1	2.83	47	95	0.1250	1.26
Fungi	Gini coefficient	0.858 (0.835–0.873)	0.880 (0.869–0.885)	0.015 (−0.010 to 0.040)	1	1.69	166	166	0.1250	0.76

Table S3. PERMANOVA with permutations restricted within campaigns, and PERMDISP

Restricted test: campaign entered as a block and the management labels were exchanged within each campaign (all 25 = 32 permutations; smallest attainable p = 0.031). PERMDISP: distances to group centroids in PCoA space, 9999 permutations.

**Marker**	**Test**	**df (num/den)**	**SS**	**R^2**	**Pseudo-F**	**p (exact)**
Prokaryotes	Management ǀ campaign (restricted)	1/4	0.0833	237	3.10	125
Fungi	Management ǀ campaign (restricted)	1/4	0.2147	151	2.53	62

**Marker**	**Factor**	**F**	**p**	**Mean distance to centroid**
Prokaryotes	Management	6.18	121	Irrigated 0.191; Rainfed 0.117
Prokaryotes	Season	0.36	628	Autumn 0.162; Spring 0.178
Fungi	Management	0.14	707	Irrigated 0.344; Rainfed 0.316
Fungi	Season	3.58	325	Autumn 0.285; Spring 0.391

Table S4. Sensitivity analyses

(a) Taxonomic rank. Two-factor PERMANOVA without the interaction (999 permutations). For fungi, the species-level fungal tables of autumn 2025 and spring 2026 contained duplicated and misassigned entries and species were therefore excluded from the analyses reported in the main text.

Marker	Rank	Taxa	R^2 management	F management	p management	R^2 season	p season
Prokaryotes	Filo	30	302	3.75	9	135	170
Prokaryotes	Clase	74	260	2.94	35	123	257
Prokaryotes	Orden	176	127	1.26	49	166	45
Prokaryotes	Género	1131	237	2.59	41	121	209
Prokaryotes	Especie	2965	264	2.94	43	109	218
Fungi	Filo	18	67	0.55	498	89	441
Fungi	Clase	41	110	0.99	366	113	343
Fungi	Orden	121	122	1.07	355	82	511
Fungi	Género	1054	151	1.38	184	85	571
Fungi	Especie	3546	272	2.75	12	36	831

(b) Excluding spring 2026 (n = 8 profiles per marker). Medians are for the remaining four campaigns; the p-value is a paired t test with df = 3.

Marker	Metric	Rainfed median	Irrigated median	Δ mean	Campaigns irrigated < rainfed	p
Prokaryotes	Richness	465.000	406.000	-14.750	2/4	0.7574
Prokaryotes	Shannon	4.446	4.504	100	1/4	0.2618
Prokaryotes	Pielou	722	745	20	0/4	0.0139
Prokaryotes	Berger–Parker	7.657	9.542	1.369	1/4	0.3569
Fungi	Richness	302.000	157.500	-139.750	4/4	0.0004
Fungi	Shannon	3.851	2.985	-809	4/4	0.0093
Fungi	Pielou	683	605	-70	4/4	0.0182
Fungi	Berger–Parker	11.156	20.309	10.785	0/4	0.0299

Marker	R^2 regime	p regime	R^2 season	p season
Prokaryotes	436	3	119	296
Fungi	276	7	150	229

Table S5. Phylum-level composition and Glomeromycota

Mean (minimum–maximum) relative abundance across the five campaign profiles per management; number of campaigns in which each phylum was higher under irrigation. No statistical test was applied.

Marker	Phylum	Rainfed, mean % (min–max)	Irrigated, mean % (min–max)	Campaigns irrigated > rainfed
Prokaryotes	Acidobacteriota	5.54 (4.13–8.77)	4.99 (3.06–10.69)	1/5
Prokaryotes	Actinobacteriota	31.33 (28.43–33.66)	24.91 (18.68–27.04)	0/5
Prokaryotes	Bacteroidota	7.61 (5.73–9.35)	8.07 (6.54–9.42)	3/5
Prokaryotes	Crenarchaeota	11.79 (9.30–14.89)	14.26 (9.93–17.63)	3/5
Prokaryotes	Firmicutes	3.45 (1.98–5.28)	8.18 (2.94–12.02)	5/5
Prokaryotes	Planctomycetota	4.64 (3.64–7.15)	4.89 (2.82–7.45)	2/5
Prokaryotes	Proteobacteria	27.28 (24.38–29.29)	24.15 (22.21–27.02)	0/5
Fungi	Ascomycota	69.04 (55.79–81.80)	66.18 (40.74–82.90)	3/5
Fungi	Basidiomycota	15.08 (9.17–20.29)	20.09 (0.68–32.44)	4/5
Fungi	Glomeromycota	0.03 (0.00–0.04)	0.00 (0.00–0.00)	0/5
Fungi	Mortierellomycota	7.32 (3.88–11.89)	6.81 (0.00–11.98)	2/5

Glomeromycota (% of ITS1 reads):

Campaign	Rainfed	Irrigated
Spring 2024	0.0047	0.0000
Autumn 2024	0.0403	0.0000
Spring 2025	0.0278	0.0000
Autumn 2025	0.0272	0.0000
Spring 2026	0.0294	0.0000

Table S6. Vineyard characteristics

Plot	Management	Cultivar	Vine age (years)	Planting density (vines ha−1)	Area (ha)	Irrigation dose (mm/ha)	Irrigation timing	Fertilization	Tillage	Plant-protection products	Sampling allocation
1	Rainfed	Pardina	>10	<3,000	5	-	-	N-P-K synthetic mineral	Shallow	Herbicides Insecticides Fungicides	Stratified random
2	Rainfed	Pardina	>10	<3,000	5	-	-	N-P-K synthetic mineral	Shallow	Herbicides Insecticides Fungicides	Stratified random
3	Rainfed	Pardina	>10	<3,000	5	-	-	N-P-K synthetic mineral	Shallow	Herbicides Insecticides Fungicides	Stratified random
4	Irrigated	Pardina	>10	>3,000	5	2.6-3.6	May to August	N-P-K synthetic mineral	Shallow	Herbicides Insecticides Fungicides	Stratified random
5	Irrigated	Pardina	>10	>3,000	5	2.6-3.6	May to August	N-P-K synthetic mineral	Shallow	Herbicides Insecticides Fungicides	Stratified random
6	Irrigated	Pardina	>10	>3,000	5	2.6-3.6	May to August	N-P-K synthetic mineral	Shallow	Herbicides Insecticides Fungicides	Stratified random
